# Green Wall Paper

**Published:** 1872-12

**Authors:** 


Beware of Green Wall Papers.—A physician in Western Massachusetts recently had a lady patient who, for several weeks, had been suffering from nausea, general prostration, and other symptoms of slow poisoning. Failing to discover the cause of the symptoms, says the "Hartford Courant," as a last resort the Doctor requested her to move from the chamber, the walls of which were covered with a very light shade of green, so light, indeed, that in the evening it could scarcely be distinguished from white. After leaving the room the symptoms immediately disappeared, and the patient rapidly recovered. A sample of the paper was forwarded for analysis to the State chemist at Hartford (Mr. Joseph Hall, of the High School), and was found to contain a large quantity of arsenic. Mr. Hall obtained the poison in the various forms of metallic arsenic, yellow tersulphite, silver arsenite, and arsenious acid or common white arsenic. He estimates that every square foot of this innocent-looking paper contained an amount of the poison equivalent to five grains of arsenious acid, or double the fatal dose for an adult person. This, in the moist warm weather of last July and August, was amply sufficient to keep the air of a room constantly impregnated with the poison, and any person occupying such a room would be as certainly poisoned as though the arsenic had been taken into the stomach. *Richmond and Louisville Med. Journal.*

